# Cannabisin F from Hemp (*Cannabis sativa*) Seed Suppresses Lipopolysaccharide-Induced Inflammatory Responses in BV2 Microglia as SIRT1 Modulator

**DOI:** 10.3390/ijms20030507

**Published:** 2019-01-25

**Authors:** Shanshan Wang, Qian Luo, Peihong Fan

**Affiliations:** Key Laboratory of Chemical Biology of Ministry of Education, Department of Natural Product Chemistry, School of Pharmaceutical Sciences, Shandong University, Jinan 250012, China; oucwangshanshan@163.com (S.W.); lqskysea@163.com (Q.L.)

**Keywords:** cannabisin F, hemp seed, neuro-inflammatory, anti-oxidative, SIRT1

## Abstract

Hemp seed (Fructus cannabis) is rich in lignanamides, and initial biological screening tests showed their potential anti-inflammatory and anti-oxidative capacity. This study investigated the possible effects and underlying mechanism of cannabisin F, a hempseed lignanamide, against inflammatory response and oxidative stress in lipopolysaccharide (LPS)-stimulated BV2 microglia cells. Cannabisin F suppressed the production and the mRNA levels of pro-inflammatory mediators such as interleukin 6 (IL-6) and tumor necrosis factor α (TNF-α) in a concentration-dependent manner in LPS-stimulated BV2 microglia cell. Furthermore, cannabisin F enhanced SIRT1 expression and blocked LPS-induced NF-κB (Nuclear factor kappa B) signaling pathway activation by inhibiting phosphorylation of IκBα (Inhibit proteins of nuclear factor kappaB) and NF-κB p65. And the SIRT1 inhibitor EX527 significantly inhibited the effect of cannabisin F on pro-inflammatory cytokines production, suggesting that the anti-inflammatory effects of cannabisin F are SIRT1-dependent. In addition, cannabisin F reduced the production of cellular reactive oxygen species (ROS) and promoted the expression of Nrf2 (Nuclear factor erythroid-2 related factor 2) and HO-1 (Heme Oxygenase-1), suggesting that the anti-oxidative effects of cannabisin F are related to Nrf2 signaling pathway. Collectively, these results suggest that the neuro-protection effect of cannabisin F against LPS-induced inflammatory response and oxidative stress in BV2 microglia cells involves the SIRT1/NF-κB and Nrf2 pathway.

## 1. Introduction

**Figure 1:**
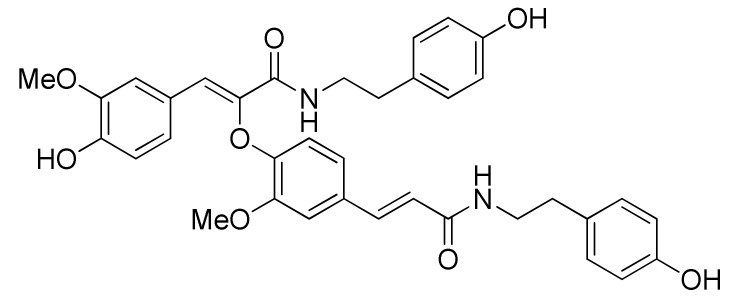
Structure of cannabisin F.

Hemp seed (Fructus cannabis, the dried fruit of *Cannabis sativa* L.) has been documented as a folk source of food for a long time [1,2]. It is growing in popularity in human nutrition as an excellent source of nutrients due to its sufficient amount and ratio of essential amino acids and fatty acids to satisfy the demand of the human diet [3,4]. Actually, hemp seed has a broad pharmacological effect in the gastrointestinal system [5], the cardiovascular system [6], the central nervous system, and the immune system [7]. Recently, hemp seed extracts were reported for their anti-aging effects and the potential to improve impaired learning and memory induced by chemical drugs in mice [8,9]. Meanwhile, recent studies showed that excluding oil and protein, hemp seed is rich in lignanamides [10,11], and that no matter hemp seed oil, protein or lignanamides all have anti-aging effect on old mice [12]. Compared with other extracts prepared by different solvents (petroleum ether, n-butanol or water), the ethyl acetate part of hemp seed demonstrates the more prominent improving effect on learning and memory ability as well as brain tissue pathological changes in experimental dementia mice [13]. According to our previous studies on hemp seed, the ethyl acetate extract contains mainly lignanamides [10,11]. It is thus reasonable to assume that lignanamides also contribute to the neuroprotective effect of hemp seed [14,15,16]. However, this was not enough involved in present literature. Continuation of our study on hemp seed provided a series of lignanamides (cannabisin A, B, C, E, F, G, etc. and other similar structures) with good antioxidant and anti-neuroinflammatory potential [10,11,15,16]. To know more about the neuroprotective effect of hemp seed lignanamides, this study selects cannabisin F (Figure 1) as representative to investigate the underlying anti-neuroinflammatory mechanism using lipopolysaccharide (LPS)-induced BV2 microglia cells, based on its good performance in a previous screening study [11].

Microglia cells are the major resident immune cells of the central nervous system (CNS). In response to external pathogen infections, cell debris or CNS injuries, microglia cells are activated quickly and release various neurotoxic and pro-inflammatory mediators such as NO, ROS (reactive oxygen species) and cytokines including interleukin-1β (IL-1β), interleukin-6 (IL-6), and tumor necrosis factor-α (TNF-α). Excessive activation will cause neuronal death and contribute to neurodegenerative processes [1,17]. Therefore, pharmaceuticals that can deliver anti-neuroinflammatory effects on microglia over-activation are considered as a reasonable and effective strategy to control neurodegenerative progression. LPS-induced BV2 microglia cells were often used as an *in vitro* anti-neuroinflammatory screening model [18,19]. LPS can induce the activation of microglia cells, thereby increasing neurotoxicity via the production of various proinflammatory and cytotoxic factors through nuclear factor kappa B (NF-κB) pathway [20]. Inflammation also triggers the generation of ROS that cause cellular oxidative damage, while microglia cells respond to the oxidative stress by accelerating inflammatory effects [21,22]. Thus, regulating oxidative stress is also a way to control the neuro-inflammatory response.

Neural cells have defense system to protect themselves from damage, and the defense system could be regulated by external stimulation. Nuclear factor erythroid-2 related factor 2 (Nrf2) is an important antioxidant sensor for cellular defense mechanisms. Once it is activated, Nrf2 translocates from the cytoplasm to the nucleus and binds to antioxidant response elements (ARE) to initiate the transcription of cytoprotective genes, such as hemeoxygenase-1 (HO-1). The transcription of these genes increases resistance to oxidative stress and displays protection against inflammation [23,24]. Nrf2 could be activated by external natural compounds [25].

The silent information regulator transcript-1 (SIRT1) is a nicotinamide adenine dinucleotide (NAD^+^)-dependent histone deacetylase that plays a significant role in anti-inflammation and anti-oxidation processes [26,27]. Previous studies showed that the activation of SIRT1 protected neurons from damage by inhibiting NF-κB transcriptional activity through the deacetylation of the p65 subunit, leading to the reduction of inflammatory cytokines [28]. SIRT1 can also inhibit the production of ROS to decrease oxidative damage [29]. Therefore, promotion of SIRT1 expression or SIRT1 activation may be beneficial in attenuating neuro-inflammation and damage due to oxidative stress.

In this study, we evaluated the neuro-protective effect of cannabisin F using LPS-stimulated BV2 microglia cells and investigated the underlying molecular mechanisms involving SIRT1 and Nrf2 modulation.

## 2. Results

### 2.1. Cannabisin F Did Not Affect the Viability of BV2 Microglia

**Figure 2:**
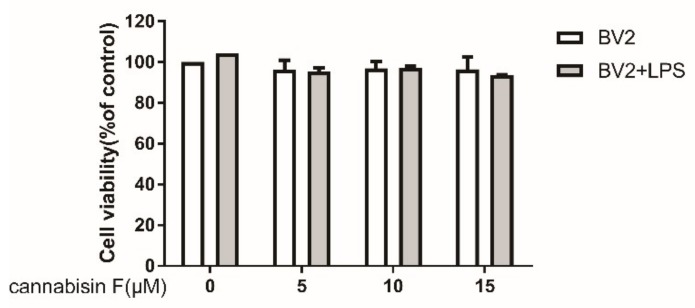
Cannabisin F at concentrations from 5 to 15 µM did not affect the viability of BV2 microglia cells with or without stimulation with LPS at 100 ng/mL for 24 h. Results are expressed as mean ± SD (Standard Deviation) from three independent experiments.

To evaluate the potential cytotoxic effect of cannabisin F in LPS-stimulated BV2 microglia cells, BV-2 microglia cells were cultured with different concentrations of cannabisin F (0, 5,10,15 µM) with or without LPS (100 ng/mL) treatment within a 24-h incubation period. Cell viability of each group was detected using the CCK-8 (Cell Counting Kit-8) kit. Figure 2 showed that treatment with various concentrations of cannabisin F had no significant effect on cell survival compared with control. Moreover, LPS (100 ng/mL) also did not affect the viability of BV2 cells in the presence of cannabisin F treatment as compared to treatment without LPS.

### 2.2. Cannabisin F Prevented the Production of IL-6 and TNF-α

**Figure 3:**
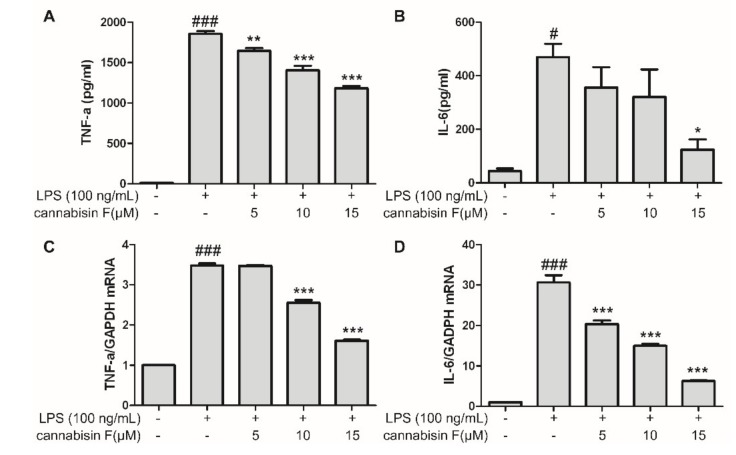
Cannabisin F inhibited IL-6 and TNF-α production (**A**,**B**) and their mRNA expression (**C**,**D**) in LPS-stimulated BV2 microglia cells. BV2 microglia cells were pretreated with cannabisin F (5, 10 and 15 µM) for 1 h and then stimulated with LPS at 100 ng/mL for 24 h. The supernatants were harvested and the secretion of TNF-α (**A**) and IL-6 (**B**) were measured by ELISA. For qRT-PCR analysis, the mRNA levels of TNF-α (**C**) and IL-6 (**D**)were determined after incubation with LPS at 100 ng/mL for 6 h. The data are presented as mean ± SD from at least three independent experiments. *p < 0.05, **p < 0.01, ***p < 0.001 as compared to the cells treated with LPS; ^#^*p* < 0.05, ^###^*p* < 0.001 as compared to the control.

LPS stimulation might induce inflammatory reaction in microglia, resulting in the release of pro-inflammatory cytokines. In order to investigate the effects of cannabisin F on the production of LPS-induced pro-inflammatory cytokines, using the ELISA (Enzyme-linked immunosorbent assay), we measured the levels of IL-6 and TNF-α released into the culture medium. As shown in Figure 3A,B, LPS stimulation alone in BV2 microglia cells significantly increased the secretion of IL-6 and TNF-α as compared to control, conversely, pretreatment with cannabisin F (5, 10 and 15 µM) decreased the levels of IL-6 and TNF-α in a dose-dependent manner as compared to LPS alone group, suggesting cannabisin F prevented the effect of LPS on the production of pro-inflammatory cytokines. Encouraged by the results, we used RT-qPCR to evaluate the effect of cannabisin F on mRNA levels of IL-6 and TNF-α in LPS-stimulated BV2 microglia cells. As shown in Figure 3C and D, LPS alone treatment elevated the mRNA levels of IL-6 and TNF-α as compared to control, pretreatment of cannabisin F (5, 10 and 15 µM) inhibited the mRNA levels of IL-6 and TNF-α in a dose-dependent manner as compared to LPS group. These results further confirmed that cannabisin F prevented the LPS-induce production of pro-inflammatory cytokines, which are involved in the inflammatory process.

### 2.3. Cannabisin F Suppressed the Production of Intracellular ROS

**Figure 4:**
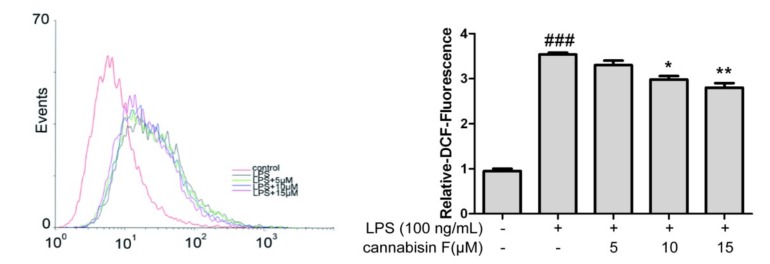
Cannabisin F inhibited the production of ROS in LPS-stimulated BV2 microglia cells. BV2 microglia cells were pretreated with cannabisin F (5, 10 and 15 µM) for 1 h prior to stimulation with LPS at 100 ng/mL and incubated for 24 h. The cells were stained with DCFH-DA for 30 min and harvested. The results are presented as mean ± SD from at least three independent experiments. *p < 0.05, **p < 0.01 as compared to the cells treated with LPS; ^###^*p* < 0.001 as compared to the control.

Previous studies revealed that ROS played a crucial role in the oxidative and inflammatory response. To determine the effect of cannabisin F on cellular ROS production in LPS-stimulated BV2 microglia cells, we evaluated the cellular ROS by the DCFH-DA (2’, 7’-dichlorofluorescin diacetate) method. As shown in Figure 4, LPS stimulation increased the cellular ROS level significantly as compared to control. However, pretreatment with cannabisin F (10 and 15 µM) suppressed the cellular ROS level as compared to the LPS group.

### 2.4. Cannabisin F Prevented NF-κB Activation and Promoted the Expression of Antioxidant Protein Nrf2/HO-1 in LPS-Stimulated BV2 Microglia Cells

**Figure 5:**
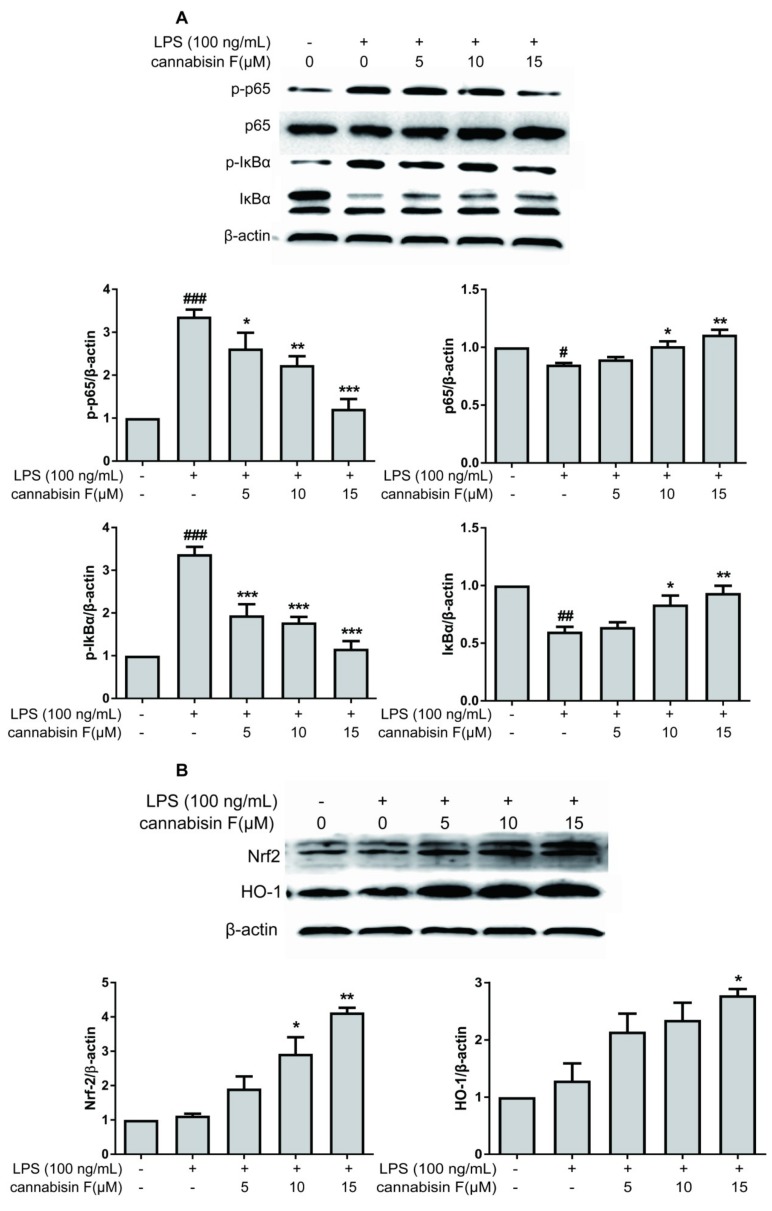
Cannabisin F inhibited the phosphorylation of IκBα, p65, but enhanced the expression of Nrf2 and HO-1 in LPS-stimulated BV2 microglia cells. (**A**) BV2 microglia cells were pretreated with cannabisin F (5, 10 and 15 µM) for 1 h prior to stimulation with LPS at 100 ng/mL for 1 h. Cell extracts were then prepared and subjected to Western blot with antibodies against IκBα, phospho-IκBα, p65, and phospho-p65. (**B**) BV2 microglia cells were pretreated with cannabisin F (5, 10 and 15 µM) for 1 h prior to stimulation with LPS at 100 ng/mL for 24 h. Cell extracts were then prepared and subjected to Western blot with antibodies against Nrf2 and HO-1. β-actin was used as the internal control for normalization. The results are presented as mean ± SD from at least three independent experiments. *p < 0.05, **p < 0.01, ***p < 0.001 as compared to the cells treated with LPS; ^#^*p* < 0.05, ^##^*p* < 0.01, ^###^*p* < 0.001 as compared to the control.

Activation of NF-κB is necessary for the expression of pro-inflammatory cytokines and enzymes. We investigated whether cannabisin F suppressed the phosphorylation of IκBα (Inhibit proteins of nuclear factor kappaB) and NF-κB p65 levels. LPS-stimulated activation of the NF-κB pathway was evaluated using Western blot analysis. As shown in Figure 5A, the result showed that phosphorylation of IκBα and NF-κB p65 levels increased in LPS-stimulated BV2 microglia cells as compared to control. However, the phosphorylation of IκBα and NF-κB p65 levels were strongly inhibited after pre-treatment with cannabisin F. Thus, these results suggest that cannabisin F has an anti-inflammatory effect in LPS-stimulated BV2 cells via inhibiting the activation of NF-κB signaling pathway.

To further understand the antioxidant effect of cannabisin F, we measured the antioxidant gene expression of Nrf2 and its downstream target gene hemeoxygenase-1 (HO-1). The results showed that cannabisin F (15 µM) can significantly increase the expression of Nrf2 and HO-1 as compared to the LPS group (Figure 5B). These data suggests that cannabisin F plays a role in antioxidant stress through regulation of the Nrf2 signaling pathway.

### 2.5. Cannabisin F Increased the Expression of SIRT1 and SIRT1 Inhibitor EX527 Reversed Anti-Inflammatory Action of Cannabisin F

**Figure 6:**
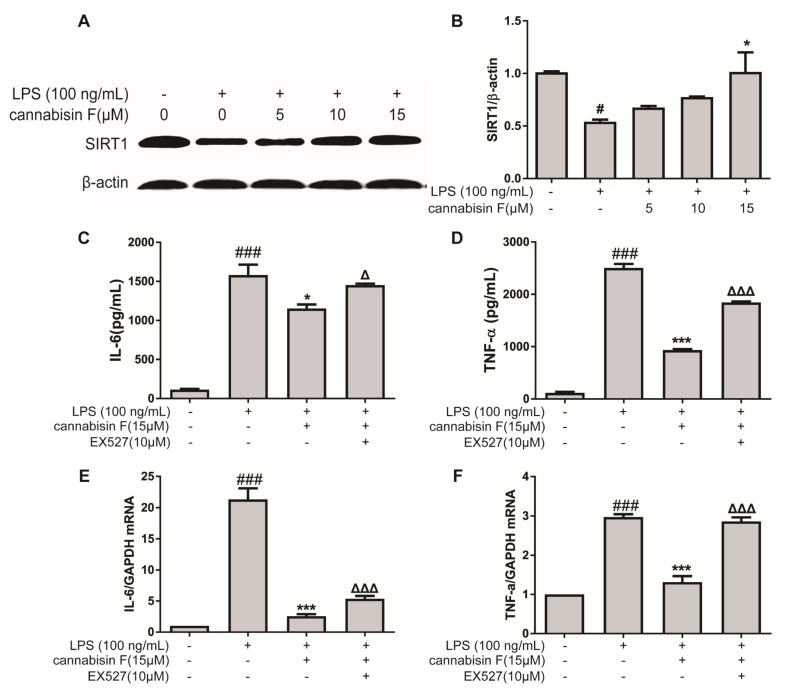
Cannabisin F increased the expression of SIRT1 and SIRT1 inhibitor EX527 reversed anti-inflammatory action of cannabisin F. (**A** and **B**) Cannabisin F enhanced expression of SIRT1. BV2 cells were pretreated with cannabisin F (5, 10 and 15 µM) for 1 h prior to stimulation with LPS at 100 ng/mL for 24 h. Cell extracts were harvested and subjected to Western blot with antibodies against SIRT1. β-actin was used as the internal control for normalization. (**C**–**F**) EX527 reversed the anti-inflammatory activity of Cannabisin F in LPS-stimulated BV2 microglia cells. BV2 cells were pre-treated with EX527 at 10 µM for 1 h, followed by treatment with cannabisin F for 1 h and then stimulation with LPS at 100 ng/mL for 24 h. Culture supernatants were harvested and analyzed for IL-6 (**C**) and TNF-α (**D**) as measured by ELISA. The mRNA levels of IL-6 (**E**) and TNF-α (**F**) were determined by qRT-PCR. The data are presented as mean ± SD from at least three independent experiments. ^△^*p* < 0.05, ^△△△^*p* < 0.001 as compared to the cells treated with LPS and cannabisin F; *p < 0.05, ***p < 0.001 as compared to the cells treated with LPS; ^#^*p* < 0.05, ^###^*p* < 0.001 as compared to the control.

SIRT1 is known to play an important role in the oxidative stress and inflammatory response. We therefore investigated the effect of cannabisin F on protein expression of SIRT1 using Western blot. As shown in Figure 6A, the result showed that LPS stimulation reduced the protein expression of SIRT1 in BV2 microglia cells as compared to control. In contrast, pre-treatment with cannabisin F attenuated the reduction of SIRT1 expression as compared to the LPS group.

To further evaluate the role of SIRT1 on the anti-inflammatory effect of cannabisin F, BV2 microglial cells were treated with LPS and cannabisin F with or without SIRT1 inhibitor EX527. BV2 cells were pre-treated with EX527 at 10 µM for 1 h, followed by treatment with cannabisin F (15 µM) for 1 h and then stimulation with LPS at 100 ng/mL for 24 h. The inflammatory cytokines were measured by ELISA. As shown in Figure 6, cannabisin F alone significantly reduced LPS-induced IL-6 (Figure 6C) and TNF-α (Figure 6D) production as compared to LPS group. However, when SIRT1 was blocked with the inhibitor EX527, the ability of cannabisin F to reduce LPS-induced inflammatory cytokines was abolished (Figure 6C–F). This finding suggests that the ability of cannabisin F to reduce LPS-induced inflammatory cytokines is SIRT1-dependent.

## 3. Discussion

Molecules that could inhibit the secretion of pro-inflammatory cytokines in microglia might help treat neurodegenerative diseases or protect the CNS from inflammatory damage. The present study contributes to research efforts to identify such natural molecules and to understand their mechanism of action. Based on our previous initial screening study [16], hemp seed cannabisins showed anti-neuroinflammatory potential in LPS-stimulated microglial cell model. The present study evaluated the neuroprotective effect of cannabisin F against neuroinflammation and oxidative stress induced by LPS. This study could contribute to establishing a global database of bioactivie constituents on hemp seed, the nutritional crop of economic and pharmaceutical importance. In addition, knowledge about the bioactive constituents excluding oils, could expand the use of hemp seed oil residue.

A number of studies showed that LPS can activate microglia cells to produce various cytokines, LPS-stimulated BV2 microglial cells were often used as an in vitro inflammatory model [30,31]. LPS-mediated neuro-inflammatory response is associated with the activation of the NF-κB signaling pathway and the increase in secretion of pro-inflammatory cytokines such as TNF-α, IL-6, and IL-1 [32]. NF-κB, a nuclear transcription factor, is known as a regulator of various genes such as iNOS, COX-2, TNF-α, IL-6, and IL-1β, which were associated with the inflammatory response [33]. In response to LPS stimuli, IκBα, an inhibitor protein of NF-κB, is phosphorylated and degraded, and thereby leading to the activation of the NF-κB signaling pathway. The translocation of NF-κB into the nucleus promotes the expression of various cytokines [34]. Here in the present study, the ELISA and qRT-PCR results showed that LPS stimulation significantly increased the secretion and gene expression of IL-6 and TNF-α in BV2 cells. Pretreatment with cannabisin F suppressed the secretion and gene expression of IL-6 and TNF-α in LPS-stimulated microglia in a dose-dependent manner. Results from Western blot analysis showed that cannabisin F effectively suppressed the LPS-stimulated protein expression of phosphorylated IκBα and NF-κB p65. Our results suggest that cannabisin F-mediated NF-κB inhibition contribute to the anti-inflammatory effect on LPS-stimulated inflammatory cytokines expression in BV2 microglial cells.

Studies reported that the activation of SIRT1 inhibited NF-κB signaling, enhanced oxidative metabolism, and inhibited inflammation. SIRT1 reduced NF-κB signaling pathways by deacetylating the p65 subunit of NF-κB complex [35,36]. In the present study, the results demonstrated that the protein expression of SIRT1 in microglia was inhibited by LPS stimulation. Consistent with these findings, a previous study reported that LPS not only inhibits the protein expression of SIRT1 but also inhibited the activity of SIRT1. The LPS-induced activation of NF-κB in this model was SIRT1-dependent [37]. Here, we found that cannabisin F inhibited the downregulation of SIRT1 induced by LPS. However, SIRT1 inhibitor EX527 significantly interfered with the inhibitory effect of cannabisin F on the production and mRNA expression of IL-6 and TNF-α in LPS-activated BV-2 cells. Therefore, the results suggest that the anti-inflammatory effect of cannabisin F resulted from its modulation on SIRT1.

In addition to the inflammatory response, LPS could also cause oxidative damage in BV2 microglia cells. The result of DCFH-DA demonstrated that LPS-stimulation increased the production of ROS microglia cells; cannabisin F could suppress this effect. Nrf2 is an important agent in the induction of various antioxidants to regulate the cellular antioxidant response against ROS. Under normal conditions, Nrf2 existed in the cytoplasm. In response to oxidative stimuli, Nrf2 transferred to the nucleus and activated downstream genes [38]. Thus, we examined the expression of Nrf2 and HO-1 protein in LPS-induced BV2 microglia cells. Our results demonstrated that cannabisin F promoted the expression of Nrf2 and HO-1 protein, suggesting that cannabisin F attenuates the oxidative stress through the modulation of the expression of Nrf2.

## 4. Materials and Methods

### 4.1. Materials

Cannabisin F was isolated from hemp seed in our laboratory [10] (Briefly, the air-dried hemp seed (5.7 kg) was crushed and defatted with petroleum ether before percolating with 75% EtOH. The 75% EtOH extract was respectively partitioned with petroleum ether, ethyl acetate, and n-butanol. The ethyl acetate extract was subjected to reverse-phase column liquid chromatography (methanol/H_2_O as eluent), MCI gel column chromatography (methanol/H_2_O as eluent), silica gel column chromatography (dichlormethan/methanol as eluent), and HPLC (methanol/H_2_O as eluent) successively, and then cannabisin F (17.21 mg) was crystallized from some fractions). Its structure was identified with Nuclear Magnetic Resonance (NMR) spectroscopy [10] and the purity was checked by high performance liquid chromatograph (HPLC) to be more than 98%. LPS from *E. coli* 0111:B4 was purchased from Sigma–Aldrich (St Louis, MO, USA). A SIRT1 inhibitor EX527 (Selisistat) was purchased from Ark Pharm (Libertyville, IL, USA). Dulbecco’s Modified Eagle’s Medium (DMEM) and the antibiotics penicillin and streptomycin were purchased from Macgene (Beijing, China). Fetal bovine serum (FBS) was from BiologiGcal Industries (Beit Haemek, Israel). CCK-8 assay kit was purchased from Dojindo (Shanghai, China). The reactive oxygen species assay kit was purchased from Beyotime Institute of Biotechnology (Shanghai, China). TNF-α and IL-6 enzyme-linked immunosorbent assay (ELISA) kits were purchased from Boster (Wuhan, China). The antibodies against NF-κB p65 and Nrf2 were obtained from Santa Cruz (Santa Cruz, CA, USA). The antibodies against IκBα, phospho-IκBα, NF-κB p65 (acetyl K310) and SIRT1 were obtained from Abcam (Cambridge, UK). The antibodies against phospho-p65, HO-1 and β-actin were obtained from Cell Signaling (Danvers, MA, USA). Trizol reagent was purchased from Takara (Takara; Shiga, Japan) and RT-PCR primers used were obtained from Sangon Biotech (Shanghai, China). Horseradish peroxidase (HRP)-conjugated secondary antibodies were obtained from ZSGB-BIO (Beijing, China). Immobilon Western Chemiluminescent Substrate (ECL) was purchased from Millipore (Billerica, MA, USA).

### 4.2. Cell Culture and Drug Treatment

Murine BV2 microglia cells were obtained from the China Infrastructure of Cell Line Resources (Beijing, China). The cells were cultured in DMEM supplemented with 10% FBS and 1% penicillin/streptomycin antibiotics and incubated in a humidified atmosphere containing 5% CO_2_ at 37 °C.

Prior to the experiments, cells were transferred to 96-well (1 × 10^4^ cells/well), 12-well (3 × 10^4^ cells/well) or 6-well (0.8 × 10^6^ cells/well) plates and incubated overnight. In all of the experiments, cells were pretreated with Cannabisin F (0, 5, 10, 15 µM) for 1 h before the addition of LPS (100 ng/mL). Cannabisin F were dissolved in DMSO at the concentration of 50 mM as a stock solution and stored at −20 °C, then diluted with the culture medium to obtain the desired final concentration before use. The final concentration of DMSO was always less than 0.4%. LPS was dissolved in sterilized PBS at the concentration of 1 mg/mL as a stock solution and stored at −20 °C, then diluted with the culture medium to the final concentration of 100 ng/mL.

### 4.3. Cell Viability Assay

The cytotoxic effect of cannabisin F on microglia cells was measured by using a CCK-8 assay kit. Briefly, the BV2 cells were distributed at a density of 1 × 10^4^ cells/well into 96-well plates and incubated overnight. Then, the cells were treated with various concentrations at 0, 5, 10, and 15 µM of cannabisin F for 1 h and co-cultured in the absence or presence of LPS (100 ng/mL) for 24 h. Thereafter, 10 µL CCK-8 dye was added into each well and the cells were incubated for 2 h. The absorbance was measured at 450 nm using a microplate reader (Bio-Rad, California, USA). Results are expressed as a percentage of the vehicle control.

### 4.4. Enzyme-Linked Immunosorbent Assay (ELISA)

The levels of TNF-α and IL-6 in the culture media were determined by enzyme-linked immunosorbent assay (ELISA) according to the manufacturer’s protocol [30]. BV2 cells at 1 × 10^4^ cells per well were seeded in a 96-well plate and incubated overnight. The cells were pretreated with 0, 5, 10, and 15 µM of cannabisin F for 1 h and stimulated with LPS at 100 ng/mL. Cell-free supernatants were collected 24 h after LPS stimulation and stored at −20 °C before analysis. TNF-α and IL-6 levels were measured with ELISA kits and absorbance at 450 nm was determined using a microplate spectrophotometer (Bio-Rad).

### 4.5. Quantitative real-time PCR (RT-qPCR)

BV2 microglia cells at 8 × 10^5^ cells per well were plated in 6-well culture plates and incubated overnight. Then, the cells were pretreated with 0, 5, 10, and 15 µM of cannabisin F for 1 h and incubated with LPS (100 ng/mL) for another 6 h. Total RNA was extracted using Trizol (Takara) and measured at 260 nm and 280 nm. The RNA (1.0 μg) obtained from BV2 cells was reverse-transcribed using the PrimeScript TMRT reagent kit (Takara) according to the manufacturer’s instructions. cDNA was used for quantitative real-time PCR (qRT-PCR). The qRT-PCR reactions were performed for 40 cycles in 10 µL reaction volumes. Samples were incubated at 95 °C for 15 s, 53 °C for 15 s, and at 72 °C for 20 s. The relative amounts of mRNA were calculated with the 2^−ΔΔ*C*T^ method. GAPDH was used as the internal control. The primer sequences were as follows: TNF-α (forward: 5′-TGAGCACAGAAAGCATGATC-3′ and reverse: 5′-TACAGGCTTGTCACTCGAATT-3′), IL-6 (forward: 5′-CCA CTTCAC AAGTCGGAGGC-3′ and reverse: 5′-CCAGCTTATCTGTTAGGAGA-3′) and GAPDH (forward: 5′-GCA GTG GCA AAG TGG AGA TTG-3′ and reverse: 5′-TGC AGG ATG CAT TGC TGA CA-3′).

### 4.6. Measurement of Intracellular Reactive Oxygen Species (ROS)

Intracellular ROS levels were determined with DCFH-DA (a stable nonpolar compound that diffuses readily into cells, intracellular ROS in the presence of peroxidase changes DCFH to the highly fluorescent compound DCF). Briefly, BV2 microglia cells were pretreated with cannabisin F (0, 5, 10, 15 µM) for 1 h and incubated with LPS at 100 ng/mL for 24 h. The cells were washed twice with PBS and incubated with 500 µL DCFH-DA (10 µM) for 30 min in the dark. After removal of DCFH-DA, the cells were digested with trypsin for 2 min at 37 °C. The serum-free medium was added into the well and the cells were transferred into a 1.5 mL microcentrifuge tube. The cells were centrifuged with 1000 rpm for 5 min and then washed twice with PBS. Fluorescence intensity of dichlorofluorescein (DCF) fluorescence as the oxidized product of DCFH was analyzed with a 488 nm excitation filter and a 525 nm emission wavelength by flow cytometry (FACS Calibur, Becton Dickinson, Franklin Lakes, NJ, USA).

### 4.7. Protein Extraction and Western Blot Analysis

The BV2 cells were washed twice with PBS, lysed on ice for 30 min using RIPA (radio immunoprecipitation assay) buffer including 1% PMSF (phenylmethanesulfonyl fluoride), and centrifuged at 14,000× *g* for 10 min at 4 °C. The protein concentrations were determined using the BCA Protein Assay Kit (Beyotime Institute of Biotechnology, Shanghai, China) according to the manufacturer’s instructions. Protein extracts were mixed with sample loading dye and heated at 95 °C for 5 min. Equal amounts of samples were separated on 8–12% sodium dodecyl sulfate polyacrylamide (SDS-PAGE) gels, and electrophoretically transferred to polyvinylidene fluoride (PVDF) membranes (Millipore, Massachusetts, USA). The membranes were blocked in blocking buffer (1× TBS, 0.1% Tween-20, 5% nonfat dry milk) for 4 h, and incubated at 4 °C overnight with primary antibodies against IκBα, p-IκBα (1:1000, Abcam), Nrf2, p65 (1:1000, Santa Cruz), phospho-p65 (1:1000, Cell Signaling), SIRT1 (1:1000, Abcam), HO-1 (1:1000, Cell Signaling), and β-actin (1:2000, Cell Signaling). The membranes were washed with TBST (Tris-buffered saline, 0.1% Tween 20) 4 times and incubated with a horseradish peroxidase-conjugated secondary antibody (1:5000, ZSGB-BIO) for 1 h at room temperature. Following 3 washes with TBST, the membranes were visualized using an enhanced chemiluminescence detection kit (Millipore). Optical density analysis of signals was performed using Image Lab software (Image lab 6.0, Bio-Rad Laboratories Inc.).

### 4.8. Statistical Analyses

Experiments were at least in triplicate. All of the data are expressed as the mean ± standard deviation (SD). The difference between treated and control cells was analyzed by one-way analysis of variance (ANOVA) using GraphPad Prism v5.0 software (GraphPad, La Jolla, CA, USA). *P*- values less than 0.05 were considered statistically significant.

## 5. Conclusions

Therefore, this study demonstrated that cannabisin F had anti-neuroinflammatory and anti-oxidative effects in LPS-stimulated BV2 microglia cells. The anti-neuroinflammatory effect of cannabisin F might be due to its modulation of the expression of SIRT1 and suppression of the activation of the NF-κB signaling pathway. Its anti-oxidative effects closely related to the promotion on the Nrf2 signaling pathway. Thus, cannabisin F may be a potential therapeutic agent for treatment of neurodegenerative diseases as modulators of SIRT1/NF-κB and Nrf2. Further study is needed to determine its specific targets of action. This study is the first to reveal the anti-neuroinflammatory and anti-oxidant effects of cannabisin F via SIRT1/NF-κB and Nrf2.

## Abbreviations

ARE	Antioxidant response elements
AD	Alzheimer’s disease
CCK-8	Cell Counting Kit-8
CNS	Central nervous system
ECL	Chemiluminescent substrate
ELISA	Enzyme-linked immunosorbent assay
FBS	Fetal bovine serum
HO-1	Heme Oxygenase-1
IκB	Inhibit proteins of nuclear factor kappaB
IL-6	Interleukin-6
LPS	Lipopolysaccharide
MyD88	Myeloid differentiation factor 88
NAD^+^	Nicotinamide adenine dinucleotide
NF-κB	Nuclear factor kappa B
NMR	Nuclear magnetic resonance
Nrf2	Nuclear factor erythroid-2 related factor 2
RIPA	Radio Immunoprecipitation Assay
ROS	Reactive oxygen species
PBS	Phosphate buffer solution
PD	Parkinson’s disease
PMSF	Phenylmethanesulfonyl fluoride
qRT-PCR	Quantitative real-time PCR
SDS-PAGE	Sodium dodecyl sulfate polyacrylamide gels
SIRT1	Silent information regulator 1
TLR4	Toll-like receptor 4
TNF-α	Tumor necrosis factor α
